# Clinical-Pathological Conference Series from the Medical University of Graz: Case No 156: 82-year-old woman with chronic diarrhea and weight loss of 20 kilograms

**DOI:** 10.1007/s00508-015-0882-8

**Published:** 2015-11-26

**Authors:** Elisabeth Fabian, Dietmar Schiller, Heimo Wenzl, Carolin Lackner, Josef Donnerer, Alexander Ziachehabi, Rene Silye, Rainer Schöfl, Guenter J. Krejs

**Affiliations:** 1 Division of Gastroenterology and Hepatology, Department of Internal Medicine III, Medical University of Vienna, Vienna, Austria; 2 Department of Internal Medicine IV, Elisabethinen Hospital, Linz, Austria; 3 Division of Gastroenterology and Hepatology, Department of Internal Medicine, Medical University of Graz, Auenbruggerplatz 15, 8036 Graz, Austria; 4 Department of Pathology, Medical University of Graz, Graz, Austria; 5 Department of Experimental and Clinical Pharmacology, Medical University of Graz, Graz, Austria; 6 Department of Clinical Pathology, General Hospital, Linz, Austria

**Keywords:** Chronic diarrhea, Sprue, Subtotal villous atrophy, Olmesartan, Drug-associated enteropathy, Adverse drug effect, Delayed hypersensitivity reaction, Hypokalemia

## Presentation of case

### Dr. D. Schiller

After two previous hospitalizations, an 82-year-old woman was readmitted with chronic diarrhea of 8-month duration and weight loss of 20 kg. This had left her so weak that she could no longer look after herself.

The watery diarrhea had started 8 months before and sometimes was worse at night. She had up to 12 bowel movements per day without abdominal pain and had lost her appetite. Duodenal biopsies revealed subtotal villous atrophy with crypt hyperplasia and increased intraepithelial lymphocytes (40 per 100 epithelial cells). Tissue transglutaminase antibodies and serum anti-endomysial antibodies were negative. Serum immunoglobulins (IgG, IgA, and IgM), thyroid stimulating hormone (TSH), differential blood count, anti-HIV antibody, ova and parasites in stool (three times), colonoscopy with multiple biopsies, capsule endoscopy of the small bowel, and abdominal computerized tomography were all negative or normal. Biopsies from ileoscopy showed changes similar to the proximal small bowel. Human leukocyte antigen (HLA)-DQ2 (positive) and DQ8 (negative) genotyping were compatible with celiac disease. Intestinal lymphocytes showed no T-cell receptor rearrangement. A strict gluten-free diet did not ameliorate the diarrhea but it improved slightly under budesonide medication. Finally, during this third hospitalization, a diagnosis of “refractory sero-negative celiac disease” was established.

Her medical history was positive for paroxysmal atrial fibrillation, hypertension, hypothyroidism following autoimmune thyreoiditis and chronic depression. Her history was negative for previous gastrointestinal complaints, foreign travel, and fever. Her family history was non-revealing.

Her medications included budesonide, amiodarone, olmesartan, amlodipine, thyroid replacement therapy (levothyroxine, liothyronine), trazodone, risperidone, and lorazepam.

On admission the patient was afebrile, body weight was 45 kg, height 162 cm; she was in poor general and nutritional condition (body mass index: 17.1 kg/m^2^). Blood pressure was 135/90 mmHg, bowel sounds were hyperactive. She appeared slightly dehydrated. Abnormal laboratory results included serum potassium 3.3 mmol/l (normal: 3.6–4.8 mmol/l), C-reactive protein (CRP) 1.5 mg/l (normal:  < 0.5 mg/l), and hemoglobin 11.1 g/dl (normal: 13.0–17.5 g/dl). All other results were negative or normal including urine dip stick, fecal occult blood, and calprotectin.

A diagnostic insight allowed an explanation for the severe diarrhea.

### Dr. C. Lackner

**Fig. 1:**
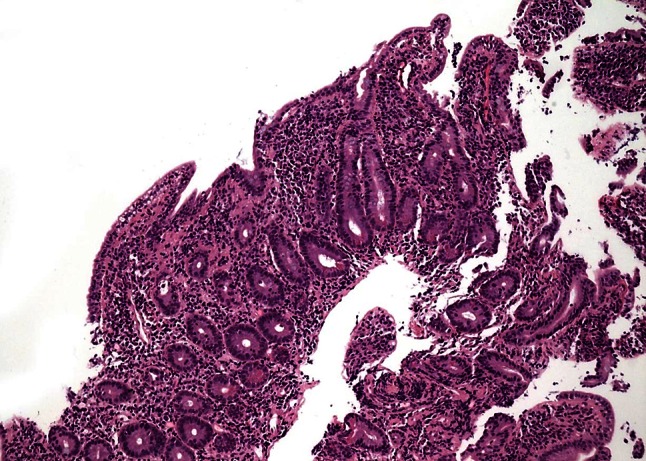
Duodenal biopsy showing subtotal villous atrophy and crypt hyerplasia (H&E, ×100)

Histological examination of the duodenal biopsies mentioned in the protocol revealed subtotal villous atrophy, chronic inflammation of the lamina propria and increased intraepithelial lymphocytes (approximately 40 per 100 epithelial cells), reactive and degenerative epithelial changes as well as crypt hyperplasia (Fig. 1). These histopathological features are not very specific and can be found with celiac disease, Crohn’s disease, enteric infection, collagenous sprue, tropical sprue, bacterial overgrowth, common variable immunodeficiency, autoimmune enteropathy, and hematological malignancies, and have also been reported as side effects of immunosuppressant drugs [1, 2]. In this case, small bowel histology is compatible with a wide spectrum of diseases.

## Differential diagnosis

### Dr. H. Wenzl

This is a case of chronic diarrhea. When a patient presents with this condition, a detailed medical history is pivotal for establishing the correct diagnosis. The patient under discussion is an elderly woman who had been suffering from chronic watery diarrhea for 8 months. She reported up to 12 bowel movements in 24 h, even at night. This important finding suggests an organic rather than functional cause for her chronic diarrhea [3]. Osmotic diarrhea can also be excluded in cases with noctural diarrhea because osmotic diarrhea always follows shortly after meals, so that it usually subsides during the night. Abdominal pain would be typical for inflammatory bowel diseases such as Crohn’s disease, irritable bowel syndrome or ischemic colitis but was not present here. The negative hemoccult and calprotectin speak against inflammatory mucosal diseases, as did the normal colon biopsies. A weight loss of 20 kg is remarkable and suggests severe bowel dysfunction including malabsorption, chronic inflammation and neoplasia, and demands extensive morphological examination. The patient underwent a pan-endoscopy including esophago-gastro-duodenal endoscopy, ileocolonoscopy with multiple biopsies, and capsule endoscopy of the small bowel. The protocol does not describe the macroscopic appearance of the duodenum and ileum, but histological assessment documented subtotal villous atrophy, chronic inflammation, and crypt hyperplasia suggesting celiac disease. In 20–25 % of patients, celiac disease first manifests itself at the age of 60 years or older [4], so based on histology alone this diagnosis could indeed be made for this 82-year-old patient. Tests for celiac disease with antibodies to tissue transglutaminase and endomysium were, however, negative. Both parameters have a high sensitivity and specificity for celiac disease [5]. Serum IgA levels were normal. Her HLA-type was DQ2 positive and DQ8 negative. In Europe, 85–90 % of patients with celiac disease are HLA-DQ2 positive and 10–15 % are HLA-DQ8 positive [6]; however, up to 30 % of the general population is also HLA-DQ2 or DQ8 positive but without the disease [7]. Celiac disease is defined as immune-mediated enteropathy with a strong genetic predisposition, which improves upon dietary exclusion of gluten [8]. Gluten is digested by luminal and brush-border enzymes into amino acids and polypeptides. The gliadin peptides induce immunological changes in the epithelium and in the lamina propria. Gliadin damages epithelial cells, leading to increased expression of interleukin-15, which in turn activates intraepithelial lymphocytes. These lymphocytes are cytotoxic and damage enterocytes expressing the surface protein MIC-A (major-histocompatibility-complex class I chain-related A). During infections or as the result of permeability changes, gliadin can enter the lamina propria, where it is deamidated by tissue transglutaminase, allowing interaction with HLA-DQ2 or HLA-DQ8 on the surface of antigen-presenting cells. Presentation of gliadin to gliadin-reactive CD4 + T cells through a T cell receptor thus results in production of cytokines that cause tissue damage, subsequently leading to villous atrophy, crypt hyperplasia, and activation and expansion of B cells that produce antibodies [7].

**Fig. 2:**
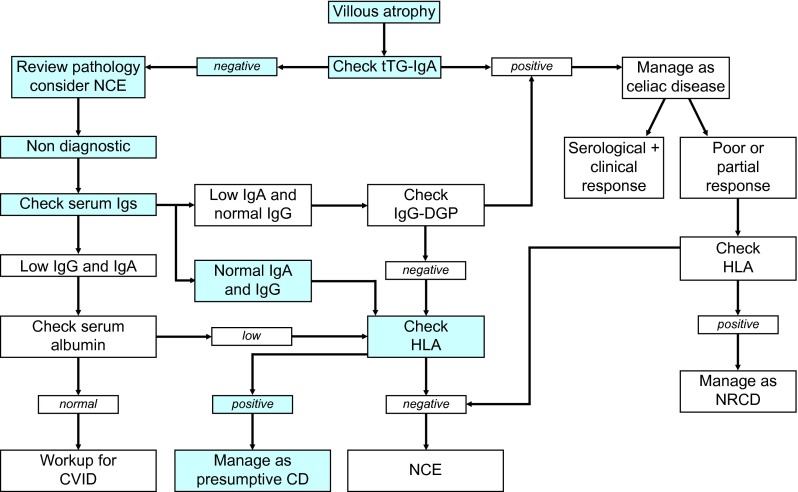
Diagnostic algorithm for small intestinal villous atrophy [15]. The diagnostic path followed with our patient is marked in *blue*. When the result (“Manage as presumptive CD”) did not satisfy the clinicians they returned to square “Review pathology, consider NCE” (*left upper corner*). Only when at this point additional other differential diagnoses were considered (see text), did her physicians arrive at the right diagnosis. *tTG* tissue transglutaminase, *Ig* immunoglobulin, *NCE* non-celiac enteropathy, *DGP* deamidated gliadin peptide, *HLA* human leukocyte antigen, *CVID* common variable immune deficiency, *CD* celiac disease, *NRCD* non-responsive celiac disease

Treatment for celiac disease is a gluten-free diet, which usually results in recovery of the mucosa and return to normal health. Our patient, however, did not respond to this diet, as is the case with 0.3–30 % of patients with celiac disease, who are subsequently diagnosed as refactory celiac disease (RCD) [9–12]. There are two types of RCD: type 1 shows a normal intraepithelial lymphocyte population, whereas type 2 displays a predominantly aberrant intraepithelial lymphocyte phenotype [13]. Taking all these findings together, our patient could have RCD type I, but I think this diagnosis has to be questioned because she is an elderly woman who might have had trouble following a strict gluten-free diet. Moreover, we speak of non-responsive sprue if patients do not respond to strict gluten-free diet for 6–12 months and of RCD if patients do not respond to strict gluten-free diet for ≥ 12 months and other causes of villous atrophy have been excluded [14]. Based on the information given in the protocol, our patient had, however, not been on a gluten-free diet long enough and as already mentioned, the elderly patient may not have been able to comply with it. Still, the negative celiac serology and the supposed non-response to a gluten-free diet urge us to consider other non-celiac enteropathies (NCE) presenting with villous atrophy. The diagnostic algorithm for small intestinal villous atrophy (Fig. 2) indicates that other etiologies of NCE have to be considered here [15]. These include common variable immunodeficiency, food allergies, tropical sprue, post viral enteropathy, eosinophilic gastroenteritis, peptic duodenitis, autoimmune enteropathy, small intestinal bacterial overgrowth, immune-mediated enteropathy, Crohn’s disease, and collagenous sprue [15]. Other diseases with small intestinal villous atrophy that merit consideration are giardiasis, HIV enteropathy, tuberculosis, radiation enteritis, Zollinger–Ellison syndrome, Whipple’s disease, intestinal lymphoma, food intolerances [16], alpha chain disease, graft-versus-host disease, hypogammaglobulinemia, severe malnutrition [5], and certain medications [17]. The medical history shows no evidence for most of these diseases; there are no further specific histopathological findings and there is no other serological abnormality. This leads us to limit the differential diagnosis to non-responsive sprue, post-gastroenteritis syndrome, small intestinal bacterial overgrowth, autoimmune enteropathy, and Zollinger–Ellison syndrome. A strict gluten-free diet over a longer period of time would show whether the patient suffers from truly non-responsive sprue. A post-gastroenteritis syndrome would be self-limiting (“watch and wait”); small intestinal bacterial overgrowth could be sought with an H_2_-breath test and analysis of duodenal aspirate, and treated with oral antibiotics; an autoimmune enteropathy could be diagnosed by an experienced gastrointestinal pathologist’s review of the biopsies and enterocyte antibodies; and in case of Zollinger–Ellison syndrome, serum gastrin would be increased, usually together with a tumor in the pancreas or duodenal wall. Actually, none of these diagnoses really seems to be likely in our patient. According to our list of potential causes for NCE, only a careful review of the patient’s medication is left, because some drugs might have adverse gastrointestinal effects (Table 1). Of the eight different medications our patient took, five document diarrhea as an adverse effect. Diarrhea is indeed a common drug-associated adverse event, and the underlying mechanisms are often unclear [18]. Diarrhea due to drug-induced enteropathy has been reported for medications such as mycophenolate mofetil [19, 20], methotrexate [21, 22], azathioprine [23] and most recently olmesartan. Among the medications taken by our patient, only one, olmesartan, is associated with villous atrophy resulting in sprue-like enteropathy. Olmesartan is an angiotensin II receptor antagonist that has been commonly prescribed for treatment of hypertension since 2002 [24]. The association between sprue-like enteropathy and olmesartan was first reported in 2012 [18]. Clinical features of this enteropathy include gastrointestinal symptoms such as diarrhea, weight loss and steatorrhea, negative IgA tissue transglutaminase antibodies (or endomysial-antibodies), evidence of enteropathy (villous atrophy) with or without collagen deposition or intraepithelial lymphocytes, lack of clinical response to a gluten-free diet, and exclusion of other causes of enteropathy [18]. Since our patient took olmesartan and presented with unexplained diarrhea, I suggest that she suffers from olmesartan-associated sprue-like enteropathy. Symptoms typically occur after olmesartan has been taken for months or years; this enteropathy can affect almost the entire gut [18], and can present a spectrum of histological damage (from normal villi to partial or total villous atrophy with variable degrees of mucosal inflammation) [25]. The mechanisms underlying olmesartan-associated enteropathy are still unclear. Interestingly, approximately 70 % of patients with olmesartan-associated enteropathy are HLA-DQ2 or HLA-DQ8 positive, which is a higher prevalence than expected in the general population (25–30 %) [26, 27]. This suggests that the presence of HLA-DQ2 or HLA-DQ8 may increase the risk for immune-mediated mucosal damage in these patients, but further studies must clarify this. Withdrawal of olmesartan intake usually results in rapid clinical and histological improvement of the enteropathy. Withdrawal of olmesartan is thus indicated for our patient.

**Table 1:** Patient’s medication and documented adverse gastrointestinal effects (*Summary of Product Characteristics*)

Medication	Gastrointestinal side effect	
	Diarrhea	Villous atrophy
Budenoside	−	−
Amiodarone	−	−
Lorazepam	−	−
Amlodipine	+	−
Olmesartan	+	+
Trazodone	+	−
Risperidone	+	−
Levothyroxine/liothyronine	+	−

## Dr. H. Wenzl’s diagnosis

Olmesartan-associated sprue-like enteropathy.

## Discussion of diagnosis

### Dr. G. J. Krejs

Dr. Schiller was the attending physician who cared for the patient and will describe further details of the patient’s course.

### Dr. D. Schiller

Arriving at a diagnosis in this case was challenging. Within a short period of time we observed a second patient with the same clinical picture and enteropathy. When we contacted Dr. Nadine Cerf-Bensussan at the Laboratory for Intestinal Immunity in Paris requesting determination of anti-enterocyte antibodies (which subsequently turned out to be negative in both patients), we were asked the decisive question of whether our patients were taking olmesartan. Discontinuation of the medication resolved the diarrhea within several days and both patients began to gain weight. Although there are many potential causes for NCE, gastroenterologists should be aware that olmesartan can cause enteropathy. Olmesartan has now been reported to be associated with sprue-like enteropathy [18, 20, 23, 25]. Our first two cases of olmesartan-associated enteropathy are currently being published as a clinical vignette in the journal “Gut” [28].

Meanwhile, a third patient presented with olmesartan-associated enteropathy at our hospital in Linz. All patients received a primary diagnosis of non-responsive/refractory celiac disease or unexplained sprue. Typical symptoms such as diarrhea and massive weight loss (10‒20 kg) always occurred after a longer period of olmesartan intake. In all patients, clinical response was rapid after olmesartan was discontinued and histological recovery was confirmed in all three patients by follow-up biopsy within 2 months. Since our patients’ recoveries were so striking and they had already been suffering for so long, we did not want to rechallenge them with olmesartan to reproduce the chronic diarrhea and villous atrophy.

### Dr. G. J. Krejs

Dr. Lackner, our pathologist, will show and explain the follow-up biopsy after the patient had been off olmesartan for 2 months.

### Dr. C. Lackner

**Fig. 3:**
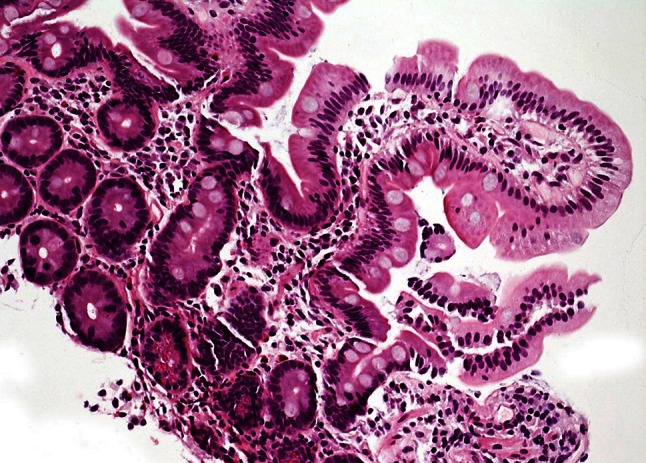
Normal villus and crypt architecture in duodenal biopsy 3 months after discontinuation of olmesartan (H&E, ×200)

Follow-up duodenal biopsy showed complete mucosal recovery. The initially described histopathological features including villous atrophy, chronic inflammation, reactive and degenerative epithelial alterations, and crypt hyperplasia were not present in the specimen after discontinuation of olmesartan. Villus:crypt ratio was within normal range (Fig. 3).

### Dr. G. J. Krejs

Currently, an association between olmesartan intake and development of sprue-like enteropathy, mainly characterized by diarrhea, weight loss and variable degrees of duodenal mucosal damage is being noted by gastroenterologists and some 90 cases have been documented worldwide. Dr. Donnerer is a clinical pharmacologist and will explain possible mechanisms of olmesartan-associated enteropathy.

### Dr. J. Donnerer

Generally, many drugs can exert adverse effects involving different organ systems and tissues. When medication is administered orally, the gastrointestinal tract is often affected by adverse events for the simple reason of being exposed to high drug concentrations. Olmesartan is an orally administered prodrug (olmesartan medoxomil) that is rapidly metabolized to the active component (olmesartan) by esterases in the gastrointestinal mucosa, portal blood and liver [29]. The mechanisms involved in olmesartan-associated enteropathy are still not fully understood.

A delayed drug hypersensitivity reaction may be the underlying mechanism leading to enteropathy under olmesartan therapy. Some medications can provoke allergic type B reactions if the pharmaceutical substance acts as a hapten and combines with a body’s own protein to form an antigen, thus inducing allergization. Generally, there are four different types of hypersensitivity reactions which can result in drug-induced allergization: type I (anaphylaxis), type II (cytotoxic effects), type III (immune-complex hypersensitivity), and type IV (T lymphocyte-mediated reactions, delayed hypersensitivity). Considering the long period of months or years between onset of olmesartan therapy and appearance of villous atrophy, it is suggested that a delayed type IV hypersensitivity reaction may cause the sprue-like enteropathy.

Some angiotensin (AT) receptor blockers are suggested to have inhibitory effects on transforming growth factor-beta 1 [30, 31], which is pivotal for maintaining gut immune homeostasis [32, 33]. Duodenal biopsies from patients under olmesartan therapy show a significant increase in the number of CD8 + cells, assuming that olmesartan-associated sprue-like enteropathy may be due to either an expansion of cytotoxic CD8 + T cells, or a dysfunctional intestinal regulatory mechanism that normally suppresses CD8 + T cell activity [34].

Another possible mechanism underlying villous atrophy in olmesartan-associated enteropathy might be a pro-apoptotic effect on intestinal epithelial cells mediated by angiotensin II. In the human gut, angiotensin II binds to two forms of receptors with different properties. AT1 receptor is expressed throughout the gastrointestinal tract; it activates growth-promoting factors and mediates major effects of angiotensin II. AT2 is mainly found in the duodenum and jejunum, and induces effects opposing those of AT1 [35, 36]. Recent studies have shown that binding of angiotensin II to AT2 promotes apoptosis of enterocytes by up-regulation of pro-apoptotic protein associated with a down-regulation of anti-apoptotic protein [37]. Moreover, in rat smooth muscle cells, drug-induced AT1 receptor blocking has been found to cause translocation of AT2 receptors from cytosol to external membrane, which may favor binding of angiotensin II to AT2 [38]. Olmesartan has a high affinity for AT1 receptors; in the case of drug-mediated saturation of these receptors, angiotensin II could increasingly bind to AT2 receptors and thus induce apoptotic effects, leading to villous atrophy without inflammation and increase in intraepithelial lymphocytes [1]. This mechanism is, however, still controversial because such effects have not been described for other angiotensin II receptor antagonists, which would be expected since interaction with the AT1 receptor is the basic therapeutic mechanism underlying this group of antihypertensives.

At present, olmesartan-associated sprue-like enteropathy may be considered an adverse effect. Since the underlying mechanisms are still unclear, more studies involving rechallenge with either olmesartan or placebo are needed. At this time a cause-effect relationship should be classified as only probable [39]. A similar enteropathy has been reported after telmisartan and irbesartan, and it is open to question whether these changes could be a class effect of sartans [40–42]. In relation to the worldwide use of olmesartan for anti-hypertensive therapy, olmesartan-associated sprue-like enteropathy occurs only rarely. However, doctors should be aware of this association when a patient under therapy with olmesartan presents with unexplained chronic diarrhea. The regulatory authorities have recently ordered that a special warning regarding sprue-like enteropathy be included in the SPC (Summary of Product Characteristics) of olmesartan medoxomil; however, since this side effect is so rare, no suggestion has been made to take olmesatran off the market.

One other comment on the medication of this patient is that she received three drugs (trazodone, risperidone, and lorazepam) that target the central nervous system (CNS). The weakness mentioned at the beginning of the protocol was probably due to her dramatic weight loss and possibly to hypokalemia, but the CNS effects of her medication should also be considered and the necessity for all the medication she is receiving should be reevaluated.

### Dr. G. J. Krejs

Dr. Wenzl, as a diarrhea expert, what do you say about the hypokalemia albeit mild in this patient? When does chronic diarrhea lead to hypokalemia? And what is your final comment on this case?

### Dr. H. Wenzl

Hypokalemia is always a sign of chronic large-volume diarrhea. The transport mechanisms in the colon try to conserve sodium but potassium has to go into the lumen in exchange. With a normal stool, 5 meq of potassium are discharged every day; with a liter of watery stool there is a potassium loss of 50 meq/l per day [43–45]. This slowly depletes total body potassium (50 meq/kg) and ultimately results in hypokalemia. Hypokalemia is prominent in pancreatic cholera or Verner–Morrison syndrome, where it is part of the name of the condition (WDHH syndrome or watery diarrhea hypochlorhydria hypokalemia syndrome) [46]. It is also observed with chronic laxative abuse. That the patient had slight hypokalemia indicates long-standing large-volume diarrhea.

This case is very interesting and emphasizes the diagnostic dilemma when patients with villous atrophy have either negative celiac serology or do not respond to a gluten-free diet. Under these circumstances, a broad spectrum of other etiologies for enteropathy has to be considered. A side effect of medication as a cause, especially when olmesartan is involved, should be part of the initial differential diagnosis of such unexplained enteropathy, as the newer guidelines now suggest [18, 47].

## Final diagnosis

Olmesartan-associated sprue-like enteropathy.
